# Ensemble fuzzy deep learning for brain tumor detection

**DOI:** 10.1038/s41598-025-90572-5

**Published:** 2025-02-19

**Authors:** Asma Belhadi, Youcef Djenouri, Ahmed Nabil Belbachir

**Affiliations:** 1 OsloMet University, Oslo, Norway; 2 Department of MicroSystems, University of South-Eastern Norway, Kongsberg, Norway; 3 Norwegian Research Center, Grimstad, Norway

**Keywords:** Computational science, Computer science

## Abstract

This research presents a novel ensemble fuzzy deep learning approach for brain Magnetic Resonance Imaging (MRI) analysis, aiming to improve the segmentation of brain tissues and abnormalities. The method integrates multiple components, including diverse deep learning architectures enhanced with volumetric fuzzy pooling, a model fusion strategy, and an attention mechanism to focus on the most relevant regions of the input data. The process begins by collecting medical data using sensors to acquire MRI images. These data are then used to train several deep learning models that are specifically designed to handle various aspects of brain MRI segmentation. To enhance the model’s performance, an efficient ensemble learning method is employed to combine the predictions of multiple models, ensuring that the final decision accounts for different strengths of each individual model. A key feature of the approach is the construction of a knowledge base that stores data from training images and associates it with the most suitable model for each specific sample. During the inference phase, this knowledge base is consulted to quickly identify and select the best model for processing new test images, based on the similarity between the test data and previously encountered samples. The proposed method is rigorously tested on real-world brain MRI segmentation benchmarks, demonstrating superior performance in comparison to existing techniques. Our proposed method achieves an Intersection over Union (IoU) of 95% on the complete Brain MRI Segmentation dataset, demonstrating a 10% improvement over baseline solutions.

## Introduction

In modern medicine, the integration of sensors has revolutionized healthcare by enabling continuous monitoring and diagnosis of diseases in diverse and complex environments^1–5^. For instance, Magnetic Resonance Imaging (MRI) and retinal imaging have become critical tools in diagnosing life-threatening conditions such as brain tumors and diabetic retinopathy. However, interpreting these medical images often requires expert knowledge, is time-consuming, and is prone to human error. Deep learning techniques have shown immense promise in addressing these challenges, as evidenced by their successful application in diagnosing COVID-19^6,7^, brain tumors^8,9^, small lesion identification^10^, and retinal diseases^11,12^.

Despite these advancements, significant barriers remain in deploying deep learning solutions in real-world clinical settings. Consider the case of brain tumor segmentation using MRI scans: accurate segmentation is critical for treatment planning, yet the imaging data is often noisy, heterogeneous, and partially labeled. A misclassification in identifying tumor boundaries could lead to incomplete resection during surgery or damage to healthy brain tissue, causing irreversible harm to the patient. Similar issues arise in other medical domains, such as retinal imaging^13^, where missed or incorrect diagnoses of retinal diseases can lead to permanent vision loss. These high-stakes scenarios highlight the need for trustworthy, interpretable, and robust deep learning and intelligent solutions that clinicians can rely on with confidence^14^.

One of the major obstacles in adopting these solutions is the inherent uncertainty in medical data, stemming from variability in imaging protocols, patient-specific differences, and limited annotations. Addressing this uncertainty is crucial for building models that can generalize well across diverse scenarios. Fuzzy deep learning has emerged as a promising approach to tackle these challenges by embedding uncertainty management into the learning process. For example, fuzzy segmentation networks for T1- and T2-weighted MRI scans employ volumetric fuzzy pooling (VFP) layers to handle noisy data and extract region-specific features with improved precision^15^. Similarly, fuzzy rough set-based models have shown promise in dealing with partially labeled datasets, optimizing weights to suppress noise and improve the reliability of learned representations^16,17^.

However, these solutions often fall short in real-world scenarios due to several limitations:

1. *Accuracy and reliability*: Current models struggle to consistently deliver high accuracy across varying data distributions, reducing their effectiveness in clinical settings.
2. *Interpretability and usability*: Most existing methods function as “black-box” systems, making it difficult for healthcare professionals to trust their recommendations, especially in high-stakes situations.
3. *Adaptability*: Existing approaches are often designed for specific datasets or imaging modalities, limiting their generalizability to diverse real-world conditions.

To address these challenges, we propose the Ensemble Fuzzy Deep Learning (EFDL) framework, a novel solution for biomedical image segmentation. This framework is specifically designed to tackle the uncertainty and variability inherent in medical imaging tasks by integrating fuzzy logic with ensemble learning. Our contributions are as follows:

1. We enhance state-of-the-art segmentation algorithms by incorporating fuzzy layers to learn and identify relevant regions in diverse biomedical applications effectively.
2. We propose a knowledge-base-driven ensemble strategy that selects the most suitable deep learning model for each image during inference, ensuring optimal accuracy and reliability.
3. We evaluate the proposed framework on widely recognized biomedical segmentation benchmarks, demonstrating superior performance in segmentation quality compared to baseline methods. While the framework significantly improves segmentation accuracy, we also identify areas for efficiency optimization.

## Related work

The objective of medical image segmentation is to locate the various labels within a given medical image. The goal of deep learning is to create models that are effective in learning the segmentation function. A medical image serves as the model’s input, and its output will be a label for each pixel in the image.

Qinghua et al.^18^ entailed the utilization of machine learning techniques for the segmentation of breast medical images with the objective of detecting cancerous lesions. Prior to preprocessing, the medical images undergo preprocessing steps such as cropping and the removal of noise, achieved through bilateral filtering, histogram equalization, and pyramid mean shift filtering. The next step involves grouping the pixels in images into super-pixels using simple linear iterative clustering. Each super-pixel has its features extracted, and two labels are made: the tumor label if the super-pixel has a tumor, and the normal label otherwise. The next step is to use the kNN classifier to determine if the pixels next to the super-pixels are tumor or normal. The tumor of the new image is eventually segmented by merging adjacent tumor super-pixels. Ghosal et al.^19^ proposed an automatic and adaptive convolutional neural network architecture designed to efficiently segment brain tumors from Multimodal Magnetic Resonance Images (MMRIs). The architecture is equipped with the ability to autonomously capture both local and global features from the input image data through interacting subpaths of varying lengths embedded within the model. To enhance its generalization capability and reduce the risk of overfitting, the model strategically incorporates dropout layers during training. Ultimately, the proposed architecture delivers precise tumor segmentation with improved efficacy. Agarwal et al.^20^ introduced a novel multi-scale dual-channel decoder for biomedical image segmentation. The proposed segmentation model employs two parallel encoders and a dual-channel decoder. The encoders, built on convolutional networks, extract features from the input images at various levels and scales. The decoder is composed of a hierarchical structure of Attention-gated Swin Transformers, enhanced with a fine-tuning strategy to improve performance.

For the segmentation of fetal medical images, a hybrid deep learning architecture was suggested in^21^. To improve the segmentation findings’ accuracy, a V-Net and attention mechanism combination is used. Instead of using batch normalization, global normalization is utilized to handle a wide range of batch sizes. To reduce the error ratio, a mixed loss function based on the dice similarity coefficient is created. Cheng et al.^22^ addressed three problems with breast lesion medical picture segmentation, including uneven breast lesion morphologies, confusing boundaries caused by comparable appearances between the lesion and non-lesion regions, and uniform intensity distributions inside the breast lesion zone. The inception of multi-scale feature maps is initiated through the application of CNN. Each layer of the CNN architecture is paired with a 1 x 1 convolutional layer in conjunction with a max-pooling operation, facilitating the detection of breast lesion boundaries. To comprehensively assess the relationship between the generated feature maps and the ultimate image prediction, the features extracted from all CNN layers are concatenated and subsequently fused using spatial-wise and channel-wise blocks. Djenouri et al.^23^ presented a brand-new, safe, cooperative platform based on augmented reality applications for biomedical segmentation. On a multi-agent system platform, distributed deep learning is carried out. A privacy-preserving approach is then created to ensure improved communication among the system’s multiple intelligent agents. In this study, a system of numerous agents is developed to simulate the group behaviors of the segmentation process. Additionally, augmented reality is used to enhance the visualization of medical patterns. To protect the privacy of the learning process, a novel privacy technique built on blockchain technology is investigated.

For diagnosing prostate cancer, a hybrid deep learning system was suggested^24^. Utilizing the Sobel filter, feature extraction is performed, followed by the application of RCNNs for the segmentation of medical images. In an innovative approach, Lin et al.^25^ introduced the Dual SWIN Transformer U-Net (DS-TransUNet) framework. This framework incorporates the hierarchical SWIN transformer into both the encoder and decoder components of the traditional U-shaped architecture. The DS-TransUNet framework leverages the advantages of self-attention computations offered by the SWIN transformer and the dual-scale encoding strategy, effectively simulating non-local interactions and capturing multiscale contexts. These features significantly enhance the semantic segmentation quality for a wide range of medical images. Luo et al.^26^ introduced a novel technique that serves as a straightforward yet highly effective method for the regularization of consistency in semi-supervised medical image segmentation tasks. The authors devised a pyramid-prediction network, inspired by the concept of a pyramid feature network, to generate a set of segmentation predictions across various scales. In order to leverage unlabeled data for the purposes of semi-supervised learning, the proposed approach focuses on minimizing the disparity between each pyramid prediction and the collective average. Furthermore, to bolster the effectiveness of the pyramid consistency regularization, the authors introduced a concept that aims to mitigate consistency loss specifically at outlier pixels, which may exhibit markedly distinct predictions from the average due to upsampling errors or a shortage of labelled data.

This brief survey of the literature reveals several shortcomings in the current medical segmentation-based treatments. The first aspect to think about while dealing with real-world situations is uncertainty. Given the fact that the developed solutions are block-box-based models, it is challenging to provide patients and medical teams with a reliable road line to follow. This may harm how these tools are used and deployed. The second drawback is that each model performs well with some types of data but poorly with others. This study investigates an ensemble fuzzy deep learning for learning for intelligent biomedical segmentation. The suggested framework is described in depth in the next section.

## Methodology

**Fig. 1:**
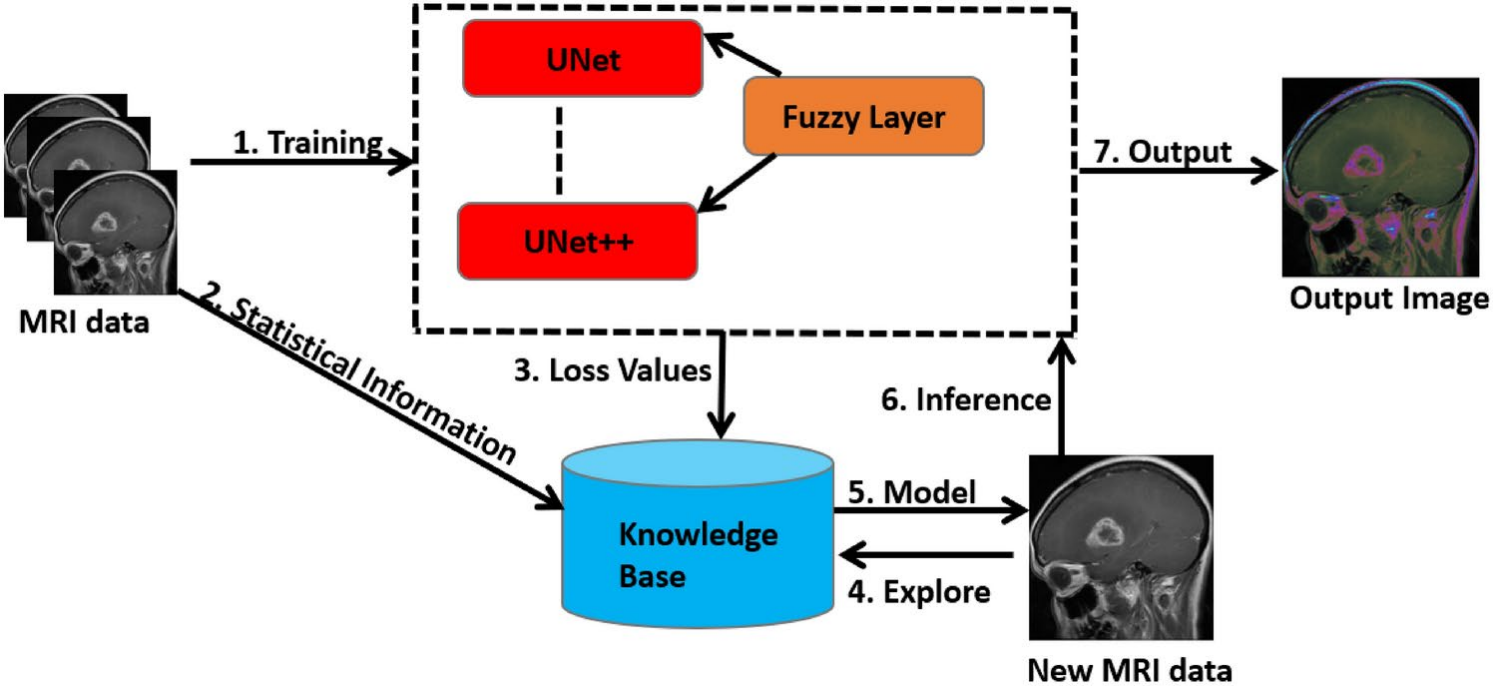
EFDL Framework: The medical sensors’ stored data are first extracted. Then, the deep learning models are trained by adding fuzzy layers. To accurately merge the results across the trained models, the relevant data from the training phase is saved in a knowledge base with ensemble learning. The fusion data is used to determine whether a model is appropriate for a certain set of test data during the inference phase.

### Overview

In this study, we introduce the Ensemble Fuzzy Deep Learning (EFDL) framework, a novel and robust approach tailored to significantly enhance segmentation accuracy in biomedical imaging, particularly under challenging conditions of uncertainty and noise. Our framework strategically integrates fuzzy logic within an ensemble of advanced deep learning models, aiming to achieve high-precision segmentation and ensure reliability even in the presence of ambiguous data. The EFDL framework synergizes U-Net-based architectures with Transformer-based models, which are refined through a fuzzy-augmented inference process to handle the unique complexities of biomedical data. Figure 1 provides a detailed visualization of the EFDL architecture, delineating the contributions of each component to the overall framework. Each aspect of the EFDL framework is rigorously formalized and theoretically validated, emphasizing reproducibility, robustness, and accuracy across diverse biomedical imaging scenarios. Leveraging the principles of ensemble learning fused with fuzzy logic, the EFDL framework assembles independent model predictions to achieve an enhanced segmentation performance. Crucially, each model in the ensemble incorporates fuzzy enhancement layers to effectively address data ambiguity and noise, culminating in a highly resilient and accurate segmentation solution. This architecture represents a substantial advance in leveraging fuzzy logic for ensemble deep learning, offering a reliable tool for complex biomedical image analysis.

#### Proposition 1

*Let* $\mathscr {M} = \{M_1, M_2, \ldots , M_n\}$ *denote an ensemble of* $n$ *deep learning models, each independently trained with fuzzy enhancement layers for biomedical segmentation tasks. We propose that the error rate of the ensemble*, $E_{\mathscr {M}}$, *is bounded by*:

1 $$\begin{aligned} E_{\mathscr {M}} \le \prod _{i=1}^n E_{M_i} \cdot (1 - \text {Cov}(M_i)), \end{aligned}$$

$E_{M_i}$ *is the individual error of model* $M_i$, *and* $\text {Cov}(M_i)$ *measures inter-model correlation*.

This error bound validates the principle of reduced ensemble error through low inter-model correlation, affirming that model diversity enhances the EFDL framework’s segmentation accuracy.

### Training phase

The EFDL framework leverages U-Net variants and Transformer-based models with integrated fuzzy layers to address data uncertainty. Below, we detail the formalized architecture and training process for each component of the proposed framework.

#### U-net-based architectures

We propose enhancing traditional U-Net-based models (U-Net, U-Net++, U-Net 3+, and NAS-UNet) with fuzzy layers to manage uncertainty in segmentation tasks. Each variant operates as an encoder-decoder network with skip connections. Given an input image $X \in \mathbb {R}^{H \times W}$, where $H$ and $W$ denote height and width, the encoder computes feature maps $F^c_i$ through convolutional and pooling layers:

2 $$\begin{aligned} F^c_i = \text {MaxPooling}(\text {ReLU}(\text {Conv2D}(F^c_{i-1}))), \forall i = 1, 2, \dots , N_c, \end{aligned}$$

The decoder subsequently reconstructs the segmentation map, achieving localization through upsampling and concatenation:

3 $$\begin{aligned} F^e_i = \text {Conv2D}(\text {Concatenate}(\text {Upsample}(F^e_{i+1}), F^c_i)), \end{aligned}$$

yielding a final output $S = \text {Conv2D}(F^e_1)$.

#### Fuzzy layer

To enhance the robustness and adaptability of the segmentation process, we propose a **fuzzy layer** that integrates the concept of **VFP**. This novel integration enables the model to dynamically handle uncertain regions in the input data, improving segmentation accuracy by emphasizing relevant features while suppressing noise. The fuzzy layer is described below.

*Volumetric Fuzzy Pooling (VFP)* The VFP operation aggregates features within local neighborhoods while preserving the structural integrity of volumetric data (e.g., 3D MRI scans). This pooling mechanism enhances spatial continuity and reduces the influence of noise. The VFP process comprises three steps:

1. *Fuzzification of Features:* The input feature map $F^c$ is transformed using the membership function:
  4 $$\begin{aligned} \mu (F^c_i) = \frac{1}{1 + e^{-\alpha (F^c_i - \beta )}}. \end{aligned}$$
  where $\alpha$ controls the steepness of the membership function, determining sensitivity to feature variations. $\beta$ defines the threshold around which membership values are centered. This function assigns a membership value $\mu (x) \in [0, 1]$ to each feature intensity *x*, which represents the degree of uncertainty associated with that feature.
2. *Weighted Aggregation:* A weighted aggregation of features is performed within a local neighborhood $\mathscr {N}(i)$. The pooled feature value $F^{vp}_i$ at position *i* is computed as:
  5 $$\begin{aligned} F^{vp}_i = \frac{\sum _{j \in \mathscr {N}(i)} \mu (F^c_j) \cdot F^c_j}{\sum _{j \in \mathscr {N}(i)} \mu (F^c_j)}. \end{aligned}$$
  This operation ensures that features with higher membership values (lower uncertainty) contribute more significantly to the aggregated output.
3. *Defuzzification:* The aggregated fuzzy features are converted back into a crisp representation, resulting in a pooled feature map that highlights high-confidence regions.

*Fuzzy-enhanced feature map* The output of the fuzzy layer combines the original feature map with the VFP-enhanced features, formulated as:

6 $$\begin{aligned} F^f_i = \mu (F^c_i) \cdot F^{vp}_i. \end{aligned}$$

This adaptive combination emphasizes features with high confidence, improving the segmentation quality in uncertain or noisy regions. The incorporation of VFP into the fuzzy layer provides several notable benefits. It reduces the impact of noisy features through weighted aggregation, particularly in complex and heterogeneous datasets, ensuring noise suppression. Additionally, the pooling operation preserves spatial and volumetric continuity, maintaining key anatomical details for structural preservation. By quantifying uncertainty through fuzzification, the layer dynamically adjusts to variations in the input data, enhancing its adaptability and robustness in handling uncertain or noisy regions. This fuzzy enhancement enables each U-Net model variant to adaptively weight uncertain regions, contributing to a more resilient segmentation output.

#### Transformer-based model

Our proposed Transformer model reinterprets the segmentation task as an object detection problem, leveraging self-attention mechanisms to perform focused region analysis in biomedical imaging. By viewing segmentation in this way, the model achieves greater accuracy in handling complex and noisy data by dynamically attending to relevant spatial regions. The core of this mechanism is the self-attention layer, which computes scores to determine the importance of each part of the image with respect to others. The attention mechanism is defined by:

7 $$\begin{aligned} \text {Attention}(Q, K, V) = \text {Softmax}\left( \frac{QK^T}{\sqrt{d_k}}\right) V, \end{aligned}$$

where $Q$, $K$, and $V$ are the query, key, and value matrices, respectively, and $d_k$ represents the dimensionality of the keys. Here, $Q$, $K$, and $V$ matrices are generated by linearly projecting the input feature map. This attention mechanism computes relevance scores between various parts of the image, assigning higher weights to areas deemed more critical for segmentation. These weights, derived from the Softmax function, enable the model to focus on critical regions, allowing for robust segmentation even in the presence of ambiguous or occluded features.

To maintain spatial dependencies - crucial for the accurate interpretation of biomedical images - we incorporate positional encodings into the Transformer model. This positional information ensures that spatial relationships within the image are preserved, allowing the model to capture both global context and localized details. The positional encoding function is defined as:

8 $$\begin{aligned} \text {PE}(pos, 2i)= & \sin \left( \frac{pos}{10000^{2i/d_{\text {model}}}}\right) , \end{aligned}$$

9 $$\begin{aligned} \text {PE}(pos, 2i+1)= & \cos \left( \frac{pos}{10000^{2i/d_{\text {model}}}}\right) , \end{aligned}$$

$pos$ denotes the position in the sequence, $i$ represents the dimension index, and $d_{\text {model}}$ is the dimensionality of the model. This encoding method provides each position in the input sequence with a unique vector that captures its relative and absolute position, effectively embedding spatial information into the model. The sine and cosine functions allow the model to learn relationships at varying scales, ensuring that it can differentiate between features based on their spatial layout.

By integrating positional encodings with the self-attention mechanism, our Transformer model adeptly balances global and local feature extraction. This dual focus enables it to detect and segment regions of interest with high precision. The positional encoding component plays a critical role, allowing the model to preserve structural integrity and spatial hierarchy, which is essential for the accurate segmentation of complex biomedical images. Together, the attention mechanism and positional encoding drive the model’s ability to perform nuanced segmentation tasks, resulting in a robust approach to biomedical image analysis.

### Model selection phase

In our proposed Ensemble Fuzzy Deep Learning (EFDL) framework, the model selection phase is guided by a structured knowledge base-driven inference mechanism. The knowledge base is constructed from a collection of feature representations and corresponding performance metrics for each image previously encountered during training. Each entry in the knowledge base contains: (i) a feature vector representing the image’s characteristics, (ii) the model used for segmentation, and (iii) the model’s segmentation performance on that image, measured using metrics such as Intersection over Union (IoU). This organization enables efficient querying and comparison during inference.

For a new test image $\textbf{X}_t$, the knowledge base is queried to identify similar images based on feature similarity. The similarity measure $d(\textbf{X}_t, \textbf{X}_i)$ is computed between $\textbf{X}_t$ and each image $\textbf{X}_i$ in the knowledge base using the Euclidean distance:

10 $$\begin{aligned} d(\textbf{X}_t, \textbf{X}_i) = \sqrt{\sum _{k=1}^{N} (x_{tk} - x_{ik})^2}, \end{aligned}$$

where $x_{tk}$ and $x_{ik}$ represent the *k*-th feature of the test image $\textbf{X}_t$ and the stored image $\textbf{X}_i$, respectively, and *N* is the number of features. Based on these distances, the *k*-Nearest Neighbor (kNN) algorithm is employed to retrieve the top *k* similar images.

Once the nearest neighbors are identified, the associated models and their performance metrics are analyzed to select the most suitable model for $\textbf{X}_t$. The selection process is guided by a combined objective function that considers both the similarity of the retrieved images to $\textbf{X}_t$ and the historical segmentation accuracy of the models. For each model $M_i$ in the ensemble $\mathscr {M}$, the loss function $\mathscr {L}(M_i, \textbf{X}_i)$ represents its segmentation performance on the retrieved images. The ideal model $M_k$ for $\textbf{X}_t$ is determined as:

11 $$\begin{aligned} M_k = \arg \min _{M_i \in \mathscr {M}} \left( \mathscr {L}(M_i, \textbf{X}_i) + \lambda d(\textbf{X}_t, \textbf{X}_i) \right) , \end{aligned}$$

where $\lambda$ is a balancing parameter that regulates the trade-off between accuracy ($\mathscr {L}(M_i, \textbf{X}_i)$) and similarity ($d(\textbf{X}_t, \textbf{X}_i)$). A higher $\lambda$ emphasizes the selection of models trained on images more similar to $\textbf{X}_t$, while a lower $\lambda$ prioritizes overall segmentation accuracy.

By dynamically adapting the selection criteria based on both image-specific features and model performance history, the knowledge base enables robust and context-aware decision-making. This structured approach enhances the EFDL framework’s capability to handle diverse biomedical imaging conditions, including variations in anatomy, imaging modalities, and noise levels, ensuring accurate and reliable segmentation across a wide range of scenarios.

### Computational complexity

#### Theorem 2

*The computational complexity of the proposed EFDL framework for each model in the ensemble is* $\mathscr {O}(n \cdot m^2)$, *where* $n$ *is the number of layers in the model and* $m$ *is the dimension of each feature map in terms of the number of neurons per side (assuming square feature maps). When utilizing an ensemble of* $k$ *models, the overall computational complexity of the EFDL framework is* $\mathscr {O}(k \cdot n \cdot m^2)$.

#### Proof

To analyze the computational complexity of the EFDL framework, we break down the complexity by examining each component within a single model’s architecture and then extend this to the ensemble of models.

1. *Single Model Complexity Analysis*: Each model within the EFDL ensemble consists of multiple layers, where the dominant layers in terms of computational cost are the convolutional and fuzzy layers. Let us consider the complexity of these layers in detail.
  Convolutional Layers: For each convolutional layer $L$ in a deep learning model, let $m \times m$ denote the dimensions of the feature map, $c_{in}$ be the number of input channels, $c_{out}$ be the number of output channels, and $k \times k$ be the size of the convolutional kernel. The computational cost of one convolutional layer with these parameters is $\mathscr {O}(c_{in} \cdot c_{out} \cdot k^2 \cdot m^2)$. If we assume that $c_{in}$, $c_{out}$, and $k$ are constants (as they do not scale with the dimensions of the feature map $m$), then the cost simplifies to $\mathscr {O}(m^2)$.
  Fuzzy Enhancement Layers: The fuzzy layer applies a membership function to each feature map element independently. The fuzzy enhancement layer thus processes each element in $\mathscr {O}(1)$ time, resulting in a complexity of $\mathscr {O}(m^2)$ per fuzzy layer.
  Self-Attention Mechanism in Transformer Models: For Transformer-based models within the EFDL framework, the self-attention mechanism involves calculating dot-product attention across elements in each feature map. With $m^2$ elements in each feature map, the attention mechanism has complexity $\mathscr {O}(m^2 \cdot m^2) = \mathscr {O}(m^4)$. However, due to the sparse structure in attention heads and optimizations like multi-head attention with dimensionality reduction, the effective cost per self-attention layer is reduced to $\mathscr {O}(m^2)$ in practice.
    For each layer in the model, the dominant operations in terms of complexity per layer are therefore the convolutional and fuzzy enhancement processes, leading to an approximate complexity per layer of $\mathscr {O}(m^2).$
2. *Total Complexity for a Single Model*: Let $n$ represent the total number of layers in the model. Summing the complexity across all layers, we get $\mathscr {O}(n \cdot m^2)$. This term represents the computational complexity of a single model in the EFDL framework.
3. *Ensemble Complexity Analysis*: In the EFDL framework, we have an ensemble of $k$ independent models. Each model operates independently and performs computations in parallel with the other models. Therefore, the total complexity for the entire ensemble is the sum of the complexities of each individual model, $\mathscr {O}(k \cdot n \cdot m^2)$.

This complexity analysis assumes that the number of input and output channels, kernel size, and attention head configurations are held constant across models, focusing solely on the dimensions $m$ and the depth $n$ of each model. Since $k$, $n$, and $m$ are the primary variables affecting computational cost, the resulting bound $\mathscr {O}(k \cdot n \cdot m^2)$ accurately represents the asymptotic complexity of the EFDL framework. This complexity analysis shows that, as feature map dimensions ($m$) increase, the computational cost scales quadratically. Thus, the EFDL framework is efficient for small to moderate feature map sizes, but additional optimization strategies (such as dimensionality reduction or sparse operations) may be necessary to maintain efficiency with very large feature maps. $\square$

**Algorithm 1:**
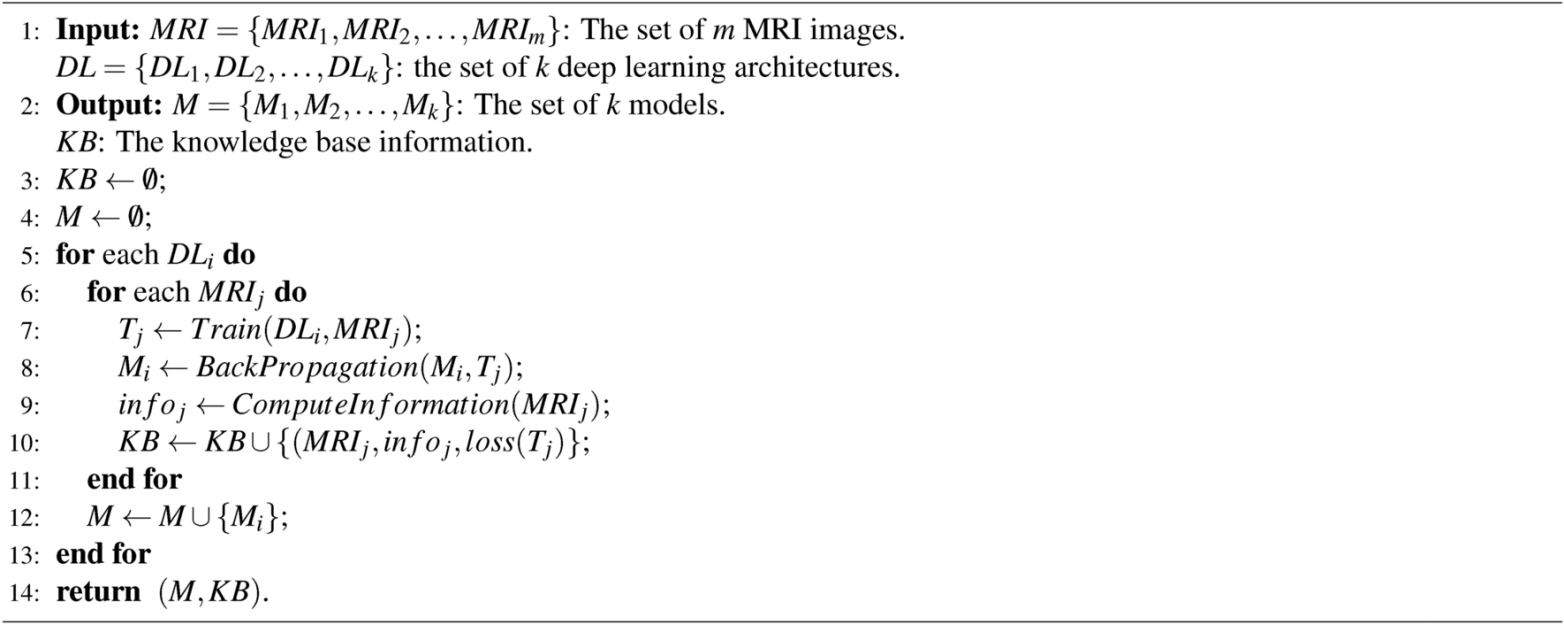
EFDL: Training

**Algorithm 2:**
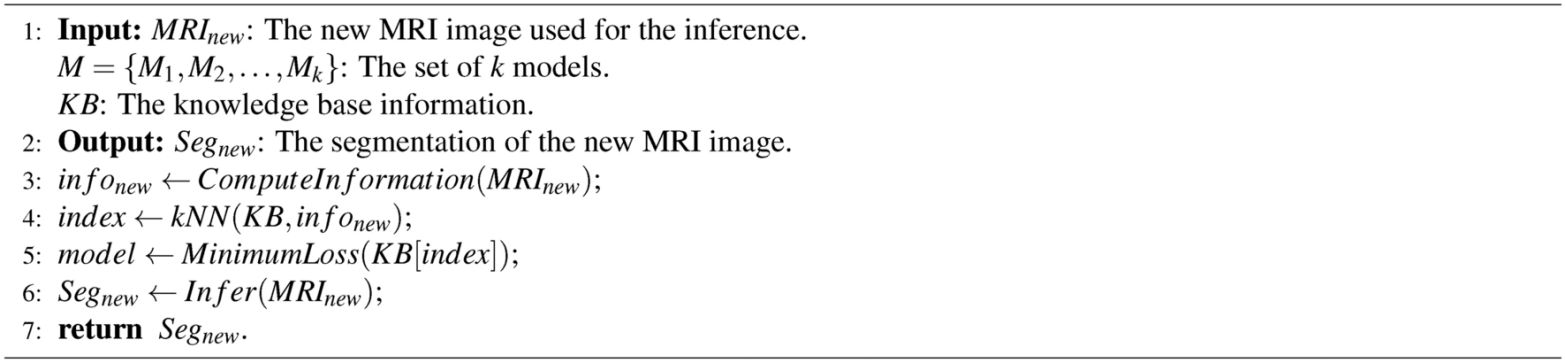
EFDL: Inference

### Pseudo code

Algorithm 1 presents the training algorithm of the EFDL. The input of the algorithm is the set of MRI images and the set of *k* deep learning architectures. The output of the algorithm is the set of *k* models and the knowledge base *KB*. The process starts by training the designing the deep learning architectures in *DL* and training the images in *MRI*. The statistical information of each *MRI* image is then computed and stored with the lost value of each model in training such an image. The models are finally updated and stored in *M*. The training algorithm returns the trained models *M*, and the knowledge base *KB*. Algorithm 2 presents the inference algorithm of the EFDL. The input of this algorithm is the new MRI image, and the output will be the segmented image. The process starts by computing the statistical information of the new MRI image. The statistical information will be used and compared with the knowledge base to first extract the most similar trained MRI image to the new MRI image, and then derive the appropriate model for this new MRI image. The derived model is then executed for this new MRI image, and the segmented image will be returned.

## Performance evaluation

**Fig. 2:**
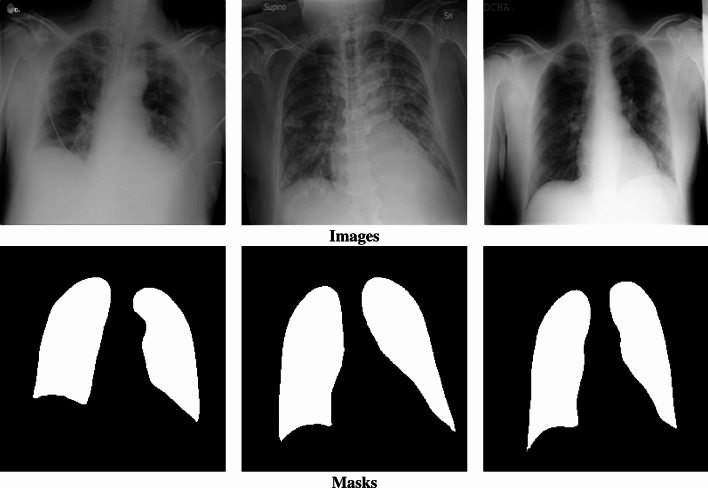
Examples from the COVID-19 Radiography Dataset, including images and their corresponding masks.

On three medical image datasets, a thorough simulation of the suggested solutions has been done. The following provides a full description of various datasets:

1. *Brain MRI* (https://www.kaggle.com/mateuszbuda/lgg-mri-segmentation): This dataset contains Magnetic Resonance Imaging (MRI) scans of the brain along with manually created FLAIR (Fluid-Attenuated Inversion Recovery) abnormality segmentation masks. The dataset includes images from 110 patients who were part of the Lower-Grade Glioma (LGG) collection in The Cancer Genome Atlas (TCGA). These patients had at least FLAIR sequencing data available, along with genomic cluster information. The images are part of a larger collection used for tumor detection and analysis, with an emphasis on identifying abnormal brain tissue in low-grade glioma cases.
2. *Liver Tumor* (https://www.kaggle.com/andrewmvd/liver-tumor-segmentation): This dataset includes 130 computed tomography (CT) scans of the liver, aimed at segmenting the liver tissue and identifying tumor lesions. The data was originally sourced from the 2017 Medical Image Computing and Computer-Assisted Intervention (MICCAI) Liver Tumor Segmentation Challenge. The dataset provides detailed CT scans, along with manual annotations to facilitate the development of automated segmentation models for liver tumor detection and treatment planning. The dataset contains various liver tumors and helps with the task of differentiating between normal liver tissue and tumor growth.
3. *COVID-19* (https://www.kaggle.com/datasets/tawsifurrahman/covid19-radiography-database): This dataset includes a collection of chest X-ray images, which cover multiple types of lung conditions, including typical pneumonia, viral pneumonia, and other respiratory diseases. It specifically contains 3,616 X-ray images of COVID-19 positive cases, 10,192 normal chest X-ray cases, 6,012 instances showing lung opacities (indicating non-COVID lung infections), and 1,345 additional images depicting cases of viral pneumonitis. The dataset also provides lung masks to assist with the segmentation of lung regions in the images. It serves as a valuable resource for building machine learning models to differentiate between various lung diseases, including COVID-19, and to aid in diagnosing and monitoring the progression of the disease based on radiographic images. Figure 2 shows examples from the COVID-19 Radiography Dataset, including images and their corresponding masks.

The overlapping labels are measured using the Intersection-over-Union (IoU) function. It counts the number of true and false labels that overlap between the model output *V* and the ground truth *U* and divides them by the total number of labels. IoU for each image $I_i$ is given as follows:

12 $$\begin{aligned} IoU_i(U_i, V_i, I_i) = \frac{|U_i \cap V_i|}{|U_i \cup V_i|} \end{aligned}$$

If and only if $IoU_i(U_i, V_i, I_i)$ is greater than a given IoU threshold then IoU $IoU_i(U_i, V_i, I_i)$ is set to 1, 0 otherwise. The IoU value for all testing images *I* is given as follows:

13 $$\begin{aligned} IoU(U, V, I) = \frac{\sum _{i=1}^{|I|}IoU_i(U_i, V_i, I_i)}{|I|} \end{aligned}$$

We evaluate EFDL against the following SOTA image segmentation techniques:

1. *FC-DenseNet*^27^: This model is considered a highly advanced solution for biomedical image segmentation. It operates by taking a single image as input and generating an output image of identical dimensions, where each pixel in the input data is associated with a corresponding label or class in the output. Its architecture consists of multiple dense layers, each intricately interconnected with the layers both above and below it, as well as with adjacent layers.
2. *DS-TransUNet*^25^: This approach endeavors to integrate both the encoder and decoder components of the conventional U-Net architecture into the hierarchical SWIN transformer framework. By doing so, it capitalized on the self-attention computations inherent to the SWIN transformer and the deliberate incorporation of dual-scale encoding. These attributes effectively replicate non-local interactions and facilitate the representation of multiscale contexts.
3. *Cross-UNet*^28^: Within the encoder phase, the model initially employed an asymmetric convolution kernel, enabling it to concurrently capture local details and the broader structural context of the source image from multiple perspectives. Subsequently, the fusion block incorporates a dual-attention mechanism, while in the decoder stage, an attention model with an extensive receptive field is utilized.
4. *FF-UNet*^29^: By modifying the receptive field through feature-fused modules and attention-gating mechanisms, it suggests few changes to the conventional U-Net architecture. The number of parameters of FF-UNet is decreased to 51% of the standard U-Net. Additionally, post-processing strategies are introduced. The contrast of biomedical datasets is enhanced using the tri-threshold fuzzy intensification-based technique.

**Fig. 3:**
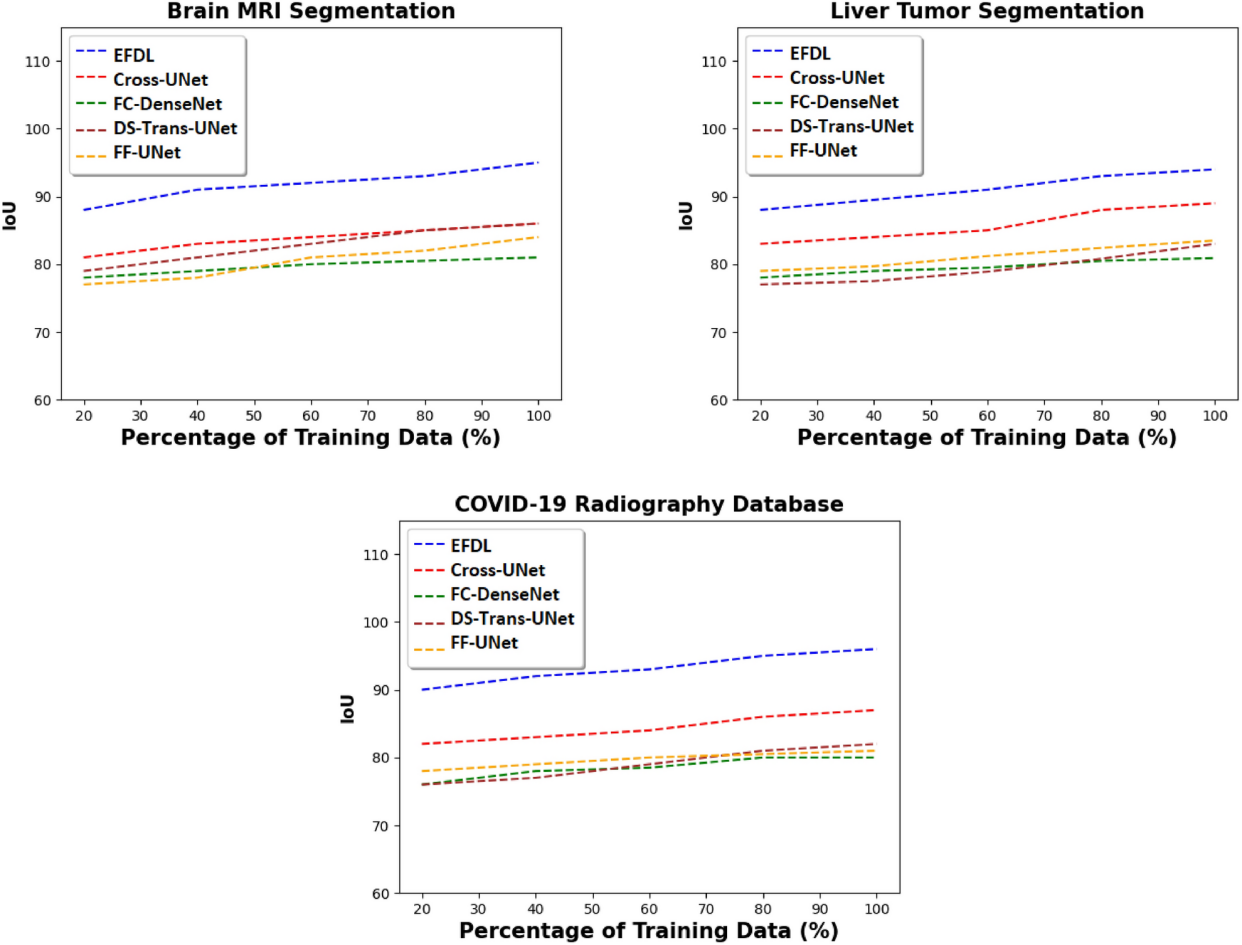
IoU of the proposed solutions and the SOTA models for different medical databases, and with fixed IoU threshold equals 0.50.

**Fig. 4:**
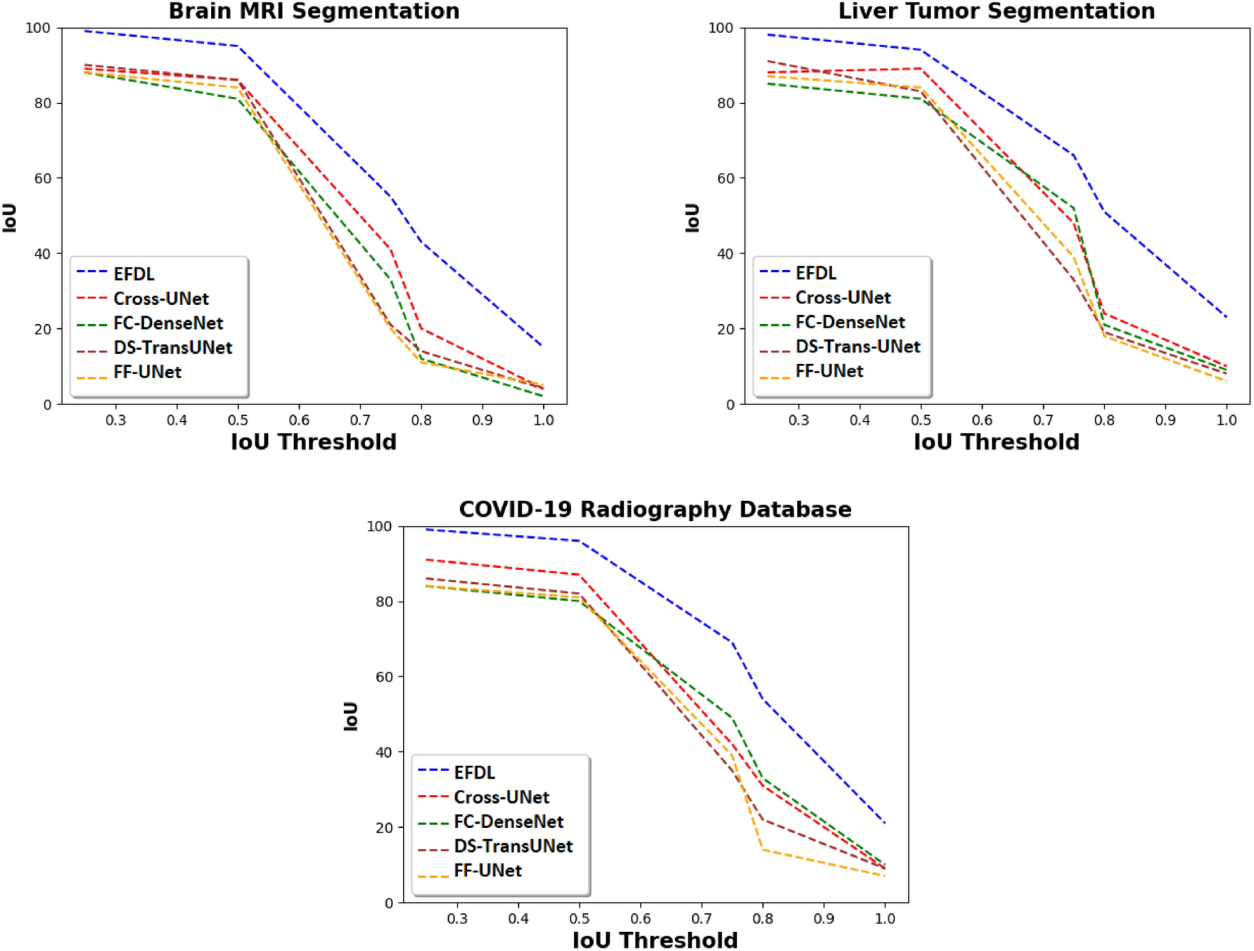
IoU of the proposed solutions and the SOTA models for different medical databases, and with different IoU threshold values.

**Fig. 5:**
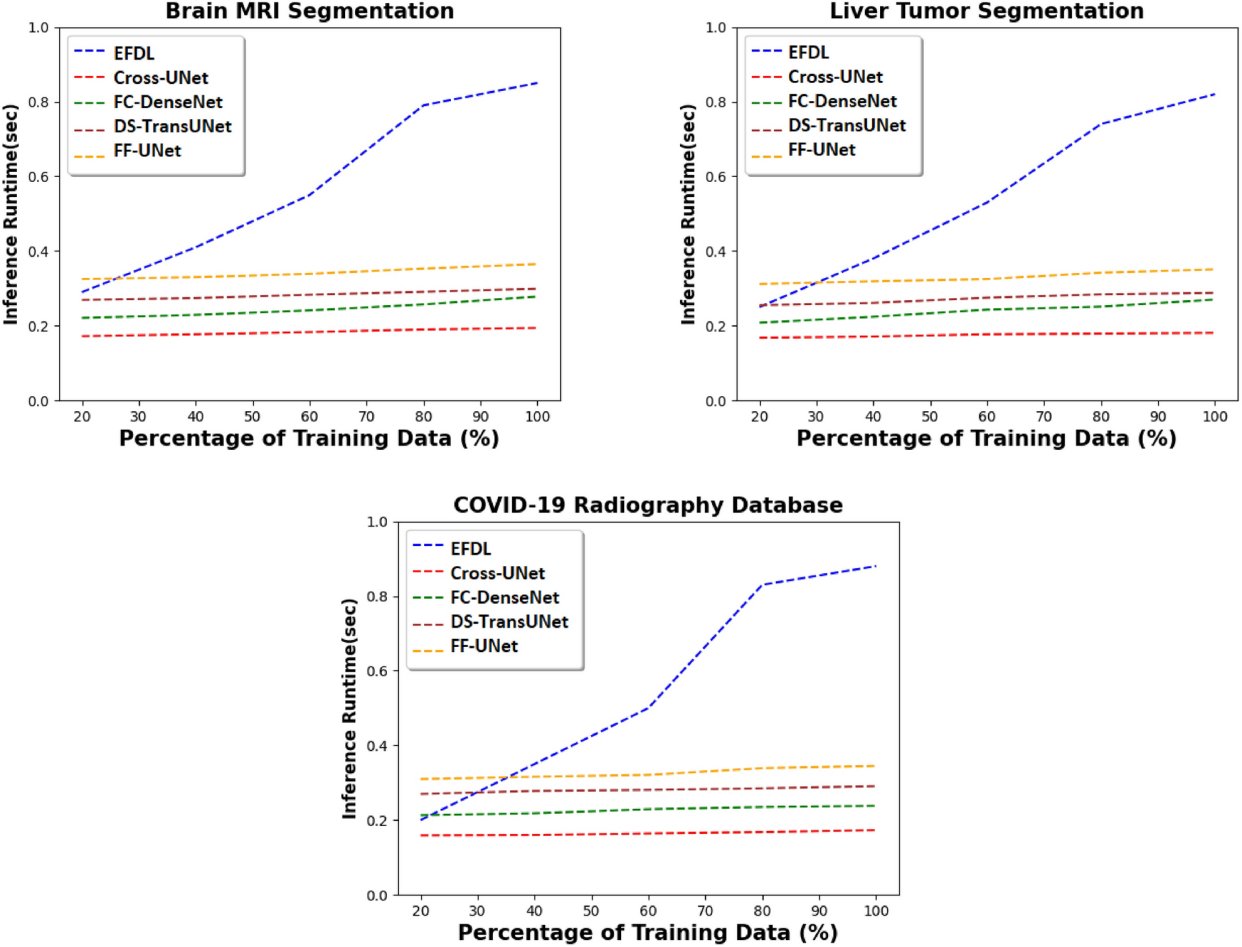
Inference runtime of the proposed solution, and the SOTA models for different medical databases, and with fixed IoU threshold equals to 0.50.

### Accuracy performance

The initial tests evaluate the EFDL’s accuracy in comparison to SOTA image segmentation techniques (FC-DenseNet, DS-TransUNet, Cross-UNet, FF-UNet) using the three datasets mentioned above. By manipulating the percentage of input images utilized, ranging from 20% to 100%, while maintaining a fixed IoU threshold of 0.50, Fig. 3 demonstrates that EFDL exhibits superior performance compared to the four baseline algorithms in the context of the union over intersection (IoU) metric. Consequently, EFDL achieves an IoU of 95% when processing the entirety of the Brain MRI Segmentation dataset. In contrast, the IoU values for the other models drop below 85% when dealing with the same configuration. In addition, Fig. 4 presents the IoU value by fixing the dataset size to 100% and varying the IoU threshold from 0.2 to 1.0. These results reveal the superiority of EFDL compared to the baseline solutions independently of the dataset used in the experiments. The IoU of EFDL is more than 20% on the Brain MRI Segmentation dataset for 1.0 as a threshold, however, the baseline solutions go below 10% for the same configuration. These results are obtained thanks to the ensemble deep learning used in the EFDL framework, where the most suitable model for each image is executed in the inference stage.

### Inference runtime performance

Using the three image segmentation datasets indicated above, the second experiment aims to assess the inference runtime of the EFDL in comparison to the benchmark image segmentation methods (FC-DenseNet, DS-TransUNet, Cross-UNet, and FF-UNet). By altering the percentage of input images used for training, ranging from 20% to 100%, Fig. 5 illustrates that EFDL exhibits longer inference times in comparison to the four baseline models. Furthermore, the disparity in computational times among the five models widens as the amount of training data employed increases. These outcomes will be able to be attributed to the inherent nature of EFDL, which operates as a consensus-based framework. This framework necessitates a negotiation strategy that involves the computation of statistical information for each testing image. Additionally, it relies on the exploration of a knowledge base established during the training phase, the size of which is contingent upon the volume of training data.

## Discussion and potential research opportunities

The proposed method will be able to explore deep learning for brain MRI analysis. Moreover, there are numerous approaches to implement incertitude when considering ideas from fuzzy learning. The clear conclusion of this paper is that fuzzy deep learning, and ensemble learning will be able to be alternative and robust ways to achieve better performance in analyzing brain MRI images. By adding the fuzzy layers, fuzzy features will be able to be determined and injected into the next layers for further learning. In addition, the use of the stored information knowledge base allows us to rapidly select the best model for each new MRI data for analysis. The main side effect of the proposed framework is that the interpretation of the model, is not straightforward and the doctors and clinicians trust the developed framework. This limitation diminishes the potential for widespread adoption of the proposed framework within hospitals and public institutions, as it may not seamlessly integrate with their existing AI tools. To address this challenge, we intend to explore more advanced interpretability solutions, such as Shapley value^30^ and GradCam^31^. Future endeavours that have the potential to yield substantial outcomes comprise: i) Anywhere in the brain will be able to have tumor dissemination. because glioma, a type of support cell that surrounds the nerves, is a mutation of the sticky cells. Support cells may be low- or high-grade gliomas, and they will be able to expand to any area of the brain because of the widespread dissemination of the virus. Using deep learning techniques, it is difficult to locate the tumor’s exact position. More advanced fuzzy deep learning^32–35^ might be used to capture the exact boundaries of the tumors. ii) Because of two issues, the series of MRI brain images have morphological uncertainty. Initially, it is important to note that each patient’s medical image exhibits variations in terms of shape and size. Additionally, the outer layer of the brain tumor exhibits variability in its structure. This is significant because fluid structures that convey valuable information about tumor shapes within their subregions will be able to be present in the outer layer of a brain tumor or inside of edema tissue. Tumor detection is one of the most difficult tasks in data learning because of the various shapes of sub-regions. More sophisticated AI strategies^36,37^ might be used to capture tumors with different shapes including small and large lesions. iii) Achieving both high resolution and high contrast is essential, as a high-contrast image contains a greater amount of information compared to an image with lower contrast in the context of MRI. MRI brain image slices typically exhibit lower quality, characterized by scattering and reduced contrast, primarily owing to image acquisition processes, including projection and tomography. Particularly, the boundaries of MRI images have poor contrast and are blurry, making it challenging to detect them. Deep learning systems face a hurdle in accurately segmenting the correct boundaries to extract enough data for additional processing to detect the tumor. Generative adversarial network^38–40^ might be used to augment the training data and therefore increase the chance of extracting the exact boundaries of the tumors. iv) Last challenge that need to be addressed for brain MRI analysis is uncertainty quantification. it is crucial for assessing the reliability of diagnostic information and treatment planning^41,42^. This could be possible by employing probabilistic models such as Bayesian frameworks allows for the estimation of uncertainty by modeling distributions of parameters rather than single point estimates. Bayesian inference provides posterior distributions that capture uncertainty in model parameters and predictions. Integrating techniques such as predictive uncertainty visualization, including heatmaps or spatial uncertainty maps overlaid on MRI images, can also provide clinicians with intuitive visual representations of uncertainty.

## Conclusion

This paper introduced a new consensus deep learning for intelligent biomedical segmentation by incorporating U-Net architectures, an intelligent approach to build a consensus, and model interpretation. The medical data is first collected from sensors. Different deep learning solutions (U-Net, U-Net++, U-Net 3+, NAS-UNet, and Visual Transformer) are then designed followed by consensus among these models. The consensus is made by creating the knowledge base containing the information of the training images with the best model for each sample. In the inference, we explore this knowledge base to efficiently deduce the best model for each testing image. Interpretation is incorporated to better understand the behaviour of the developed framework. Several tests on the actual use case of the biomedical segmentation process have been done to verify the effectiveness of the proposed framework. The acquired results show that our recommended methodology is effective in biological health informatics contexts. For future perspectives, we plan to improve both the learning process and in particular the inference runtime processing. For instance, proposing new strategies to determine the image information or an efficient strategy to explore the consensus knowledge base. Exploring other case studies such as tumour detection, automatic disease diagnosis, epidemic outbreak prediction, and drug discovery is also on our near agenda.

## Data Availability

The datasets generated and/or analysed during the current study are available in the Kaggle repositories: 1. https://www.kaggle.com/andrewmvd/liver-tumor-segmentation. 2. https://www.kaggle.com/mateuszbuda/lgg-mri-segmentation. 3. https://www.kaggle.com/datasets/tawsifurrahman/covid19-radiography-database.
